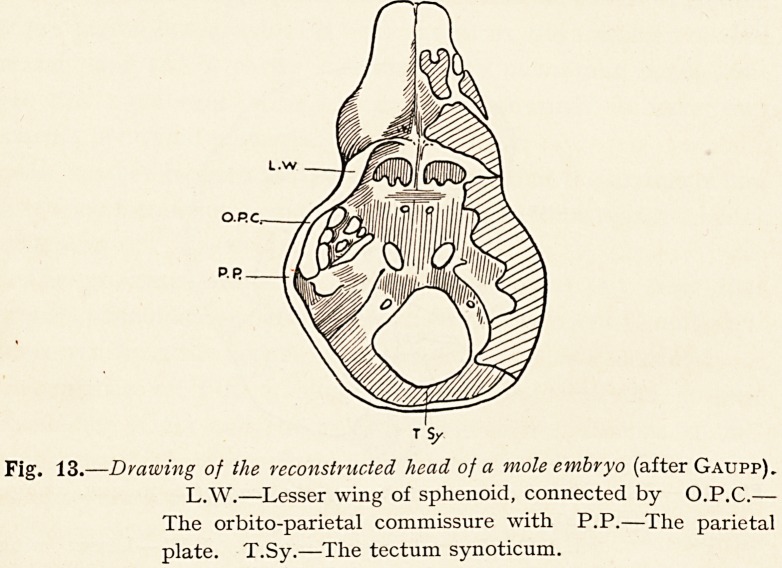# The Long Fox Lecture: The Development of the Human Skull

**Published:** 1910-06

**Authors:** Edward Fawcett

**Affiliations:** Professor of Anatomy and Dean of the Medical Faculty, University of Bristol.


*"Scire est nescire, nisi id me
Scire alius sciret."*

JUNE, 1910.

INDEX THROUGH

### THE LONG FOX LECTURE:

THE SIXTH ANNUAL LECTURE ARRANGED BY THE COMMITTEE OF THE LONG FOX MEMORIAL,
DELIVERED IN THE MEDICAL LIBRARY OF THE UNIVERSITY OF BRISTOL ON DECEMBER 12TH, 1909.

J. MICHELL CLARKE, M.D., F.R.C.P., *Pro-Vice-Chancellor, in the Chair.*

BY

EDWARD FAWCETT, M.D.,
*Professor of Anatomy and Dean of the Medical Faculty, University of Bristol.*

ON

### THE DEVELOPMENT OF THE HUMAN SKULL.

YOU are assembled not so much for the purpose of hearing me deliver this lecture, but for the purpose of keeping green the memory of one who was as honoured a member of our profession as ever trod the boards of the room in which we are gathered.

By the kind invitation of your committee, the onus of delivering the lecture founded in honour of Dr. Long Fox has devolved on myself, and I propose in this lecture to deal with some embryological subjects on which I have worked for some years, and which more particularly concern the development of the human skull.

Few subjects have engaged the attention of so many workers as this which I have chosen, and the fact that some two hundred and fifty observers have worked at and written on it is, I think, a sufficient guarantee that the last word has not been said on the subject. Many of the old-day researchers have been handicapped by the methods of research available to them; and taking that into account, it is surprising that many of them have been as accurate as they have been in their conclusion, but naturally many errors have arisen.

Since the introduction of the wax-plate method, introduced by Born and improved on by Strasser, these errors are reduced to a minimum, and although the results obtained can only really be guaranteed to represent the conditions present in that embryo from which they were obtained, they are at least accurate. It may interest you to know how the models before you are made.

Serial sections of embryos or parts of embryos are cut to a convenient thickness, say 10 to 20 microns, and these are projected by means of a lantern microscope on to paper; paper of a very common type which will be readily absorbent is best. This paper, which is obtained in rolls, can be conveniently suspended, very much as a towel is suspended, and pulled out for serial drawings to be made on it. The projected image is arranged to be a certain magnification of the section, say 50 diameters, for a reason which I will explain later. The parts of the image which it is wished to model are then carefully drawn by means of a B crayon, which gives a good, dead-black image, and can be readily seen in a bright light, a great advantage over the ordinary lead pencil.

The drawing having been made, the paper containing it is cut off—a whole series may be drawn from the roll of paper, to be divided up later—and it is then placed face downwards on a lithographic stone which has previously been well moistened with turpentine, and if possible warmed. The paper is carefully flattened out to express air bubbles, then on the sides of the paper, at a convenient distance from the drawing, brass strips of a known thickness are laid, say 1 mm. Hot beeswax is then poured on the paper between the strips and rolled out with a hot metal roller, the roller not being hot enough to scorch the beeswax.

The melted beeswax is then allowed to congeal, and another piece of paper is laid upon it, and this is carefully rolled until it comes to a level with the brass strips. We now have obtained a beeswax sandwich 1 mm. in thickness, which is just fifty times as thick as the section from which it is made. A 1 mm. plate is very convenient to cut and work with. The wax plate thus made is then placed under pressure for a time, and later is hung up to dry. When dry it is ready for use. All parts to be modelled are cut nearly completely around with a sharp knife held nearly vertically. They are not completely isolated, but kept connected by bridges with neighbouring parts of the future model. All other parts are cut away, the general result being like a piece of fretwork. Each plate is treated in the same way. The plates are then piled up one on the top of the other until as many as desired are in position. The superimposed edges are then welded together with a hot spatula, and what bridges are unnecessary are cut away with a sharp knife. The model so obtained is smoothed off and painted with one colour. This, very shortly, is the method which we know as Strasser's modification of the Born reconstruction method.

Before it is possible to understand the general process of growth of the skull, I must recall to your minds one or two fundamental facts of vertebrate structure. You all know that all vertebrates possess superimposed an alimentary canal, a notochord and a neural tube. You will remember, too, that by the side of the notochord masses of mesoblast appear, which gradually surround it and the nervous system. Were we dealing with the ordinary body region, which is simplest, we would say that the part which grew around the notochord and the nervous system formed the primordial membranous vertebral column. This becomes segmented at a later date, and chondrified to form the primordial cartilaginous vertebral column, chondrification taking place from the neighbourhood of the notochord in a dorsal direction to form ultimately a cartilagious tunnel for the spinal cord, which subsequently becomes ossified.

The general process is the same, with some restriction, in the region of the head. In the head region we have to deal with a neural tube, a notochord, and an alimentary canal (the pharynx), in that order dorso-ventrally. The notochord extends from that region which will become the permanent vertebral column as far as that up-growth from the primitive mouth which helps to form the pituitary body, but it is strikingly different from the notochord of the vertebral column in its relation to the primordial cartilaginous vertebral column. In the vertebral column this notochord runs centrally through the vertebræ, but in the skull it is seen to perforate the hinder region of the basilar part from above downwards, run underneath the middle part of the basilar region, and ascend through the anterior part of that region. For many reasons the hinder part of this basilar region, which ultimately becomes the occipital bone, is regarded as a part of the vertebral column which has been commandeered by the skull.

In the head region the membranous primordial capsule spreads from the region of the notochord around not only the neural tube or brain, but around the various sense organs connected with it, viz. the olfactory, the visual and the auditory, and the alimentary canal or pharynx. This part of the primordial capsule, save in the occipital region, gives no hint of segmentation, and there are probably many reasons why it should not be segmented, for as Hertwig tells us the skull undoubtedly arose in water-inhabiting animals, it was advantageous to have an unsegmented front-piece to act as a "cut-water," the means of locomotion in fishes being by powerful contraction of the trunk muscles. Further, it is necessary to have a fixed point from which the muscles moving the feeding apparatus can act; and finally, with the advancing importance of one extremity of the nervous system, it is important that the skeleton which encloses it shall not be a movable one.

That part of the membranous primordial capsule which surrounds the neural tube (brain) is conveniently termed the primordial membranous neuro-cranium, whilst that which surrounds the pharynx is termed the membranous-visceral cranium. Both membranous neuro-cranium and visceral-cranium are at first practically uniform, but later thickenings take place in them, as pointed out by Levi, which thickenings correspond in great part with those parts which will become converted into cartilage, and ultimately into bone. These membranous thickenings do not all appear at the same time: the first to appear is the occipital, then follow the sphenoidal and the auditory capsule, and lastly the ethmoidal.

The membranous neuro-cranium having been established, chondrification next follows, leading to the formation of what is termed the primordial neuro-chondro-cranium.

*The primordial neuro-chondro-cranium.*—Before an intelligent view of the neuro-chondro-cranium can be obtained it is necessary to call to mind the conditions holding good in lower animals.

First let me state that in that lowly fish, the amphioxus, the skull is entirely membranous, the notochord forming a very important element in the floor of that cranium, and reaching forwards to its anterior extremity.

In the cartilaginous fishes the cranium is an almost complete cartilaginous box, but with the advance in size of the brain the cartilaginous box becomes less and less complete. The greater part of the roof of the cranium is formed by membrane, cartilage appearing only at the base of the cranium, the sides, and between the two halves in the form of a narrow cartilaginous bridge, as you will see in the reconstructed model of the head of the 30 mm. embryo (Fig. 1).

Let us now see how this cartilaginous primordial cranium grows—first in lower types, then in man.

Chondrification commences at the base in all types, in the neighbourhood of the notochord. In the lower types a pair of cartilages arises between the auditory vesicles and the notochord; these, from their relationship to the notochord, are known as the parachordals. In front of these, and enclosing between them the ethmoidal element of the pituitary body, arise two other cartilages called the trabeculæ; these unite together anteriorly, and spread out to form the ethmoidal plate, which is separately connected with the organ of smell. Posteriorly the trabeculæ

unite with the parachordals, which previously have united with one another around the notochord (Fig. 2) to form the basilar plate, and this basilar plate encloses the notochord as far forwards as the back of the pituitary body.

In man the arrangement is very different. The notochord certainly is traceable forwards as far as what is early recognised as the dorsum sellæ in the immediate neighbourhood of the pituitary body, but there are no distinct parachordal cartilages; further, the basilar plate is laid down behind the pituitary body in two segments, a more posterior and a more anterior, the two

**Fig. 1.**—*An early stage in the chondro-cranium of a lower vertebrate.* N.E.—The nasal epithelium. A.—Optic vesicle. T.R.—Trabecula. O.—Auditory vesicle. P.C.—Parachordal. N.—Notochord. P.B.—Pituitary body.

**Fig. 2.**—*A late stage in a similar vertebrate, in which the parachordals have fused with one another and with the fused trabeculæ.* E.P.—Ethmoidal plate. N.O.—Nasal organ. A.—Optic vesicle. T.R.—Trabecula. P.C.—Parachordal. O.—Auditory vesicle. N.—Notochord. B.P.—Basilar plate.

being separated by a connective tissue interval, as first noticed by Kölliker (Fig. 3). There is, according to Levi, no justification for the description of trabeculæ. When the sphenoidal and occipital segments of the basilar plate have fused together, it will be seen that the relationship to the notochord is very different from that which has been previously described. The notochord, instead of running centrally through the basilar plate, enters it from above and behind after leaving the apex of the cartilaginous dens (odontoid process); then it sinks through the basilar plate, to run on its under surface in close contact with the epithelium

**Fig. 3.**—*Photomicrograph of a sagittal section of the head of a 15 mm. human embryo* (Dr. BARKER).

B.P.—Basilar plate. C.T.—Connective tissue bridge. S.—Sphenoid. P.—Pituitary body. N.C.—Notochord perforating basilar plate. T.—Tongue. L.—Larynx.

**Fig. 8.**—*Photomicrograph of a coronal section of a 19 mm.* (HARVARD) *embryo.*

A.O.—Is upper part of the orbital or lesser wing.

of the roof of pharynx ; later it rises up through the cartilage of the basilar plate to end in the dorsum sellæ behind the pituitary body (Fig. 4).

The further process of growth of the chondro-cranium is of great interest. Various masses of cartilage appear independently at different periods—not, as was formerly thought, all at the same time. As my own work has chiefly concerned the sphenoidal region, I propose to deal more especially with it. The sphenoidal region is that part of the basilar plate in the neighbourhood of the pituitary body, and it may conveniently be

**Fig. 4.**—*Drawing of a reconstruction of the basilar plate of a 24 mm. embryo.* A.—Axis vertebra. D.—Odontoid or dens. B.P.—Basilar plate. N.C.—Notochord. Sr.—Dorsum sellæ, or posterior clinoid processes. P.—Pituitary body. S.—Anterior part of corpus sphenoidale.

divided into a pre- and a post-pituitary part. You all know that appended to the sphenoid in the adult are parts which go by the name of lesser and greater wings ; pterygoid plates, internal and external (lingulæ) ; and small bones in front of the body, called sphenoidal turbinates. Until comparatively recently it has been customary to regard the wings of the sphenoid as outgrowths from the cartilaginous body of the sphenoid.

It will be shown that this, as Levi and others have also pointed out, is not the case. Let us consider first of all the case of the lesser wings, or alæ orbitales. These, already formed in the membranous primordial cranium, commence to chondrify outside the optic nerve ; the nucleus of chondrification increases in size, growing backwards behind the optic nerve until it reaches the pre-pituitary part of the corpus sphenoidale, forming thus the hinder wall of the optic foramen ; at a later period the nucleus extends inwards in front of the optic nerve, and completes the optic foramen in front. As chondrification advances, the lesser wing becomes of enormous size, extending outwards beyond the orbit into what one knows later as the temporal fossa, in which it ends in a sharp point. In the drawing of a model of the sphenoid of a 19 mm. embryo (Fig. 5) the orbital wing (O.W.) is

**Fig. 5.**—*Drawing of a reconstructed sphenoid of a* 19 *mm. embryo.*
O.W.—Lesser wing of sphenoid. P.A.—Processus alaris. T.W.—Greater wing of sphenoid. C.S.—Corpus sphenoidale.

seen in a pro-cartilaginous condition, extending almost vertically upwards from the corpus sphenoidale (C.S.), and about its centre a nucleus of cartilage is visible, especially well shown on the left side as an oval light patch.

In the drawings of another model of a 19 mm. embryo, lent by Professor Minot, of Harvard (Fig. 6), one sees the lesser wing from above, consisting of a curved mass of cartilage bending in behind the optic nerve, to reach but not fuse with the corpus sphenoidale (M.P.). An almost better impression is got of the condition by looking at the model from below (Fig. 7), where A.O.

is the orbital or lesser wing, and N.O. is the optic nerve. It is clear, then, that the lesser or orbital wing is developed quite independently of the body, as seen in the models represented.

**Fig. 6.**—*Drawing from above of a reconstruction of the sphenoid of a* 19 *mm. embryo* (Harvard Collection).

O.W.—Lesser wing of sphenoid. O.W1.—That limb of the lesser wing which closes behind the optic foramen. S.R.—The independent dorsum sellæ. P.A.—Processus alaris, or lingula.

**Fig. 7.**—*Drawing of the same model as Fig.* 6, *from the under side.*

A.O.—Lesser or orbital wing of sphenoid. N.O.—Optic nerve. A.T.—Greater wing perforated by N.T.—The second division of the fifth nerve. P.A.—Processus alaris. A.C.—Auditory capsule. N.C.—Notochord.

If one examines a photomicrograph of a section of the embryo used in building up the model (Fig. 8) one can see, A.O., the lesser wing apparently separated into two parts by a gap, the upper part being oval in form, the lower nearly circular, and the lower circular part is separated from a central mass, the corpus sphenoidale, by a dark line—perichondrium. From the outer half of the lesser wing of the sphenoid there grows

**Fig. 9.**—*A drawing of a model of the head of a* 30 *mm. embryo.*

A.—Lesser wing of sphenoid-anterior wall of optic foramen. P.—Posterior wall of optic foramen. S.E.C.—Spheno-ethmoidal cartilage. A.O.—Outgoing process of lesser wing towards P.P.—The parietal plate. A.T.—The greater wing of the sphenoid, or ala temporalis L.N.P.—Lateral nasal process of nasal capsule. O.P.—Connective tissue forming outer wall of orbit.

forward over the eyeball a plate of cartilage (Fig. 9), which at its inner end becomes connected with the nasal capsule. Its inner end is separated by a gap from the inner end of the lesser wing—the orbito-nasal fissure—a fissure through which the nasal nerve enters the orbit. This plate of cartilage, which is especially well

**Fig. 10.**—*Photomicrograph of a coronal section of a 19 mm. embryo* (Harvard Collection), *showing the independence of the greater wing.*

A.T.—Greater wing. II.5.—Second division of the fifth nerve. C.S.—Corpus sphenoidale. P.A.—Processus alaris.

**Fig. 11.**—*Photomicrograph of a coronal section of the head of an* 80 *mm. embryo, showing the cartilage of the great wing.*

A.T.—Undergoing ossification. E.P.—Is membrane bone forming the external pterygoid plate. If this be traced upwards it will be found to be continued into membrane bone which forms that part of the sphenoid which lies in the temporal fossa.

seen in the 30 mm. embryo, subsequently disappears. It is known as the spheno-ethmoidal cartilage (Fig. 9. S.E.C.).

At this period it is interesting to note that the so-called lesser wing is enormously greater than the greater wing.

Let us now consider the greater wing, or as it is often called the ala temporalis, though that is not a good name. This like the lesser wing is developed quite independently. It is represented in the drawing of the model of a head of a 19 mm. embryo of my own collection (Fig. 5, T.W.), and in the drawing of an embryo of similar length lent by Professor Minot. In Fig. 7, A.T., the greater wing or ala temporalis (T.W., Fig. 5) is seen to contain above its middle a nucleus of cartilage which lies below a nerve marked in the drawing by a light circle. The nerve is the second division of the fifth; all that part which is cross hatched is unchondrified. The greater wing is seen to be connected by connective tissue with a stalk which projects outwards from the corpus sphenoidale. This stalk is known as the processus alaris or lingula (Fig. 5, P.A.). This independent origin of the greater wing is by now well known. The photomicrograph shows it (A.T., Fig. 10) perforated by the second division of the fifth nerve, connected with the processus alaris (P.A.) by a dark perichondrial sheet.

It apparently has been assumed that this greater wing in course of time extends into the temporal fossa, to form that part of the sphenoid found in the temporal fossa,—but I have shown elsewhere[1] that this is not the case; moreover it has been understood that it grew backwards to enclose the third division of the fifth nerve and the middle meningeal artery. That, however, is not the case. It has also been assumed that it formed a considerable part of the outer wall of the orbit, that part known as the orbital plate of the great wing; that again is certainly not the case. The cartilage of the great wing forms little more than the circumference of the foramen rotundum and the pterygoid process. The statement that the lesser wing is greater than the greater wing in fœtal life is correct, but it must be clearly understood that the statement is applicable only to the cartilaginous condition.

[1] *Anatomischer Anzeiger* and *J. Anat. and Physiol.*

In Fig. 9, which is a drawing of part of the head of a 30 mm. embryo, the greater wing (A.T.) is seen at the floor of the orbit having the appearance of a bent forefinger, holding in its concavity (actually perforated by it) the second division of the fifth nerve. Above it is seen a light quadrate area (marked O.P.), which is the anlage of the orbital plate, but it is formed of connective tissue only.

It has been assumed further that the external pterygoid plate of the sphenoid is preformed in cartilage which has grown down from the greater wing. That, however, is not the case. A glance at the photomicrograph (Fig. 11) shows us the cartilage of the greater wing (A.T.) partly surrounded by membrane bone, and that this membrane bone has grown downwards to form the external pterygoid plate (E.P.).

As has before been said, then, it is clear that the cartilaginous great wing forms but a small part of that part known as the bony great wing.

The processus alaris or lingula, which runs out from the corpus sphenoidale is also chondrified independently, not by outgrowth from the corpus sphenoidale as generally assumed.

A glance at Fig. 5 shows us that between the corpus sphenoidale (C.S.) and the greater wing (T.W.) there is an independent nodule of cartilage, which is marked P.A. This is the processus alaris or lingula.

The dorsum sellæ also arises quite independently, as seen at the 19 mm. stage. This is a condition noticed first by Fischer in the macaque, but so far as I know has not hitherto been so described to man. In Figs. 6 and 4, of which Fig. 6 is drawn from the sphenoid of a 19-mm. embryo, the dorsum sellæ is seen as a transverse bar of cartilage (S.R.), lying one inch behind the pituitary body. In Fig. 4 the same cartilage is seen in section (S.R.), and it forms a round mass placed independently on the basilar plate just behind the pituitary body. To the anatomist this independent chondrification of the dorsum sellæ is of great interest, because it explains a condition met with occasionally in the adult skull. When ossified this bar of cartilage forms the posterior clinoid processes, and they add great depth to the pituitary fossa; but every now and then one meets with a very shallow pituitary fossa, and the posterior clinoid processes are absent. Gruber has recorded a suture between the posterior clinoid processes and the rest of the sphenoid, showing that they can be independently ossified, and it is quite possible that the condition present in the 19 to 24 mm. stages explains the independent ossification of the posterior clinoid processes. Further, if one suppose that such independent ossification takes place, and that no synostosis follows, then during maceration the posterior clinoid processes become lost, and the pituitary fossa remains flat.

**Fig. 12.**—*Side view of a reconstruction of the head of a 30 mm. embryo* (Bryce).

L.N.P.—Lateral nasal process. L.W.—Lesser wing. P.P.—Parietal plate. A.C.—Auditory capsule.

During chondrification of the sphenoid the auditory capsule chondrifies, first around the semicircular canals and vestibule, and later around the cochlea. This is, perhaps, what one might expect, as the cochlea is evolved later than the semicircular canals, moreover it is phylogenetically late in acquiring its coils.

From the cartilage enclosing the semicircular canals and vestibule of the ear a curious process or plate of cartilage is developed (Fig. 12), a process which was first noticed by Spöndli, but has escaped the notice of our English text-books. This process is a forwardly directed plate of cartilage (P.P.) which partly occupies the place where the parietal bone will be developed, and it is known as the parietal plate. This parietal plate is an exceedingly interesting structure, because it is the posterior moiety of what was in lower types the orbito-parietal commissure which ran continuously from the outer end of the lesser wing of the sphenoid to the auditory cartilage (Fig. 13).

The anterior part of this orbito-parietal commissure is the lesser wing of the sphenoid. In man the great increase in size of the brain has interrupted this continuity. This parietal

**Fig. 13.**—*Drawing of the reconstructed head of a mole embryo* (after Gaupp). L.W.—Lesser wing of sphenoid, connected by O.P.C.—The orbito-parietal commissure with P.P.—The parietal plate. T.Sy.—The tectum synoticum.

plate at a later date disappears altogether, as does a great part of the lesser wing of the sphenoid.

Behind the parietal plate there appears a transversely directed bridge of cartilage, which connects the vestibular cartilage of one side with that of the other, and on that account is called the tectum synoticum.

It is of great interest to note that from this tectum synoticum there is directed a small ascending process, which is seen in reptilia (amphibia), but so far as I know has not been seen in birds and fishes. Between the tectum synoticum the two occipital arches of cartilage grow in course of time towards one another, but they remain separated for a considerable period by a triangular plate of connective tissue or membrane, the spino-occipital membrane. The tectum synoticum is for a time the only part of the cranial vault formed by cartilage.

The ethmoidal region is late in chondrifying, later than any of the other median parts. The ethmoidal plate forms the septum of the nose, and anteriorly sends outwards lateral expansions, which in all likelihood are the lateral nasal cartilages. The remaining lateral walls of the nasal capsules are chondrified independently, and form at the same time the outer wall of the nose and the inner wall of the orbit. By the side of the lower end of the fore part of the nasal septum two small cartilages, the paraseptal cartilages, are developed. These cartilages are otherwise known as the Jacobsonian cartilages. From the outer wall of the nasal capsule there grows backwards, at about the 30 mm. stage, a small process of cartilage known as the lateral nasal process (Fig. 12, L.N.P.), and I think first described by Mihalkovics. It was for long believed to be the anterior remnant of the long-lost palato-pterygo-quadrate bar of cartilage of lower types, which forms to a large extent the maxillary arcade of lower vertebrates. Sutton, in a paper in the *Proceedings of the Zoological Society,* figures such a bar of cartilage as extending from the nasal capsule behind to the malleus behind in a portion of the third month. There is certainly, in my experience, no evidence in favour of such bar existing ; it does not even exist in birds.

From the mass of details with which I have troubled you let us evolve some more general statements. We have proceeded through a membranous primordial neuro-cranium and a cartilaginous primordial neuro-cranium, and we have seen that this primordial neuro-cranium, as Kölliker and others thought, is not developed " wie aus einem Gusse," but is formed by independent masses of cartilage appearing at different times ; and not only are these cartilages formed at the base of the skull, but they appear at the sides and vault. Nevertheless, the amount of cartilage developed at the sides and roof is small in comparison with the whole neuro-cranium, and, speaking broadly, has diminished in proportion to the increase in size of the brain.

Now let me conclude by thanking you all for your patient endurance of what must have proved a very dry subject for you; and may I also express my warmest thanks to Mr. Stuart Stock for all the help he has given me, without which this lecture would have been impossible; further, to Professor Minot, of Harvard, Professor Bryce, of Glasgow, and many old pupils for the material on which the work was done.

## Figures and Tables

**Fig. 1. Fig. 2. f1:**
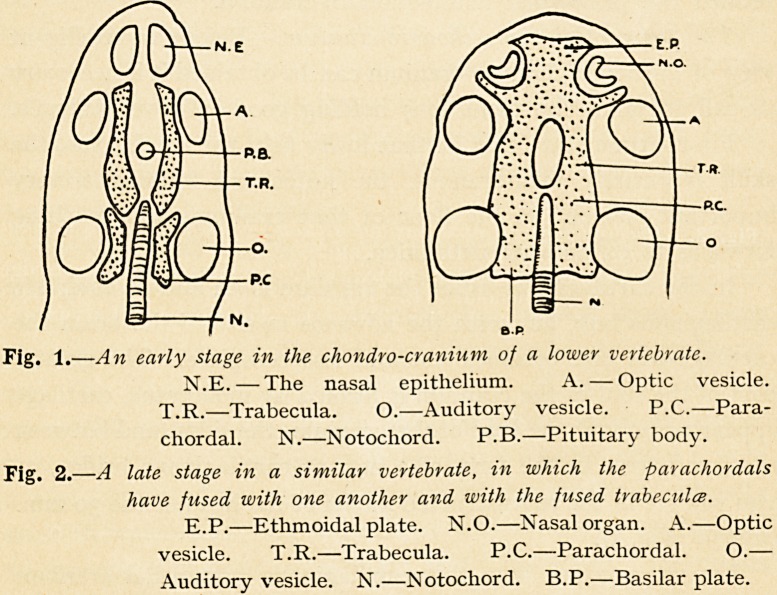


**Fig. 3. f2:**
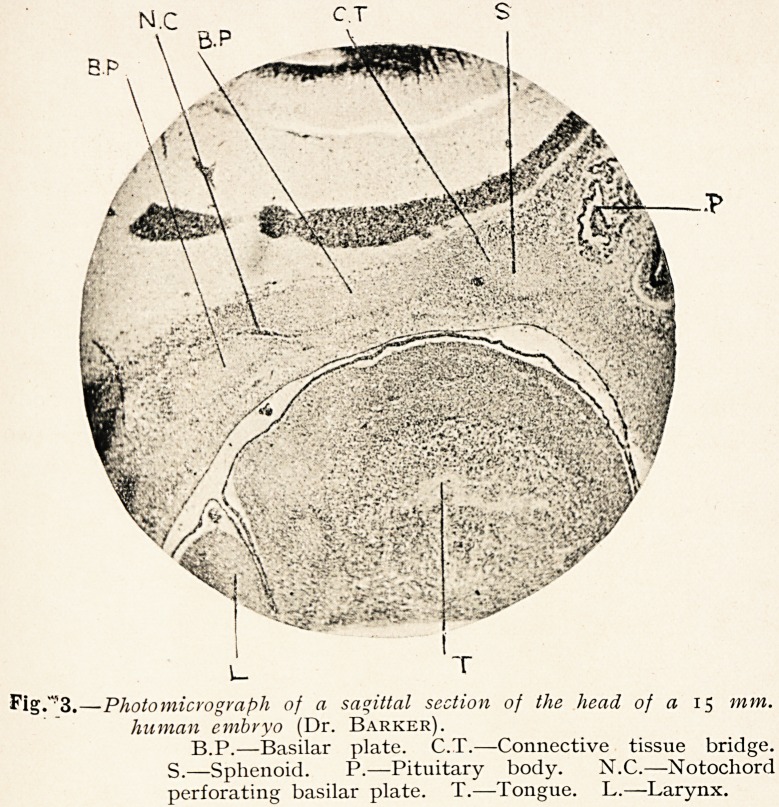


**Fig. 8. f3:**
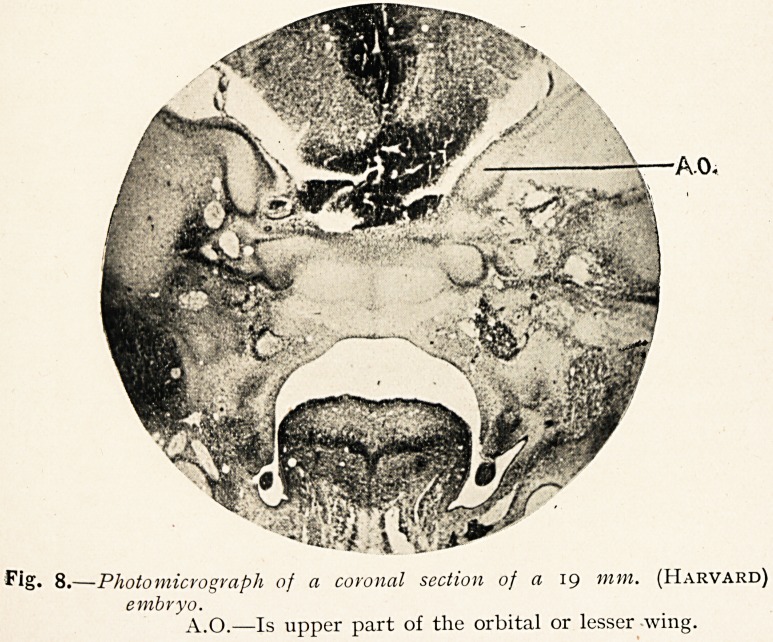


**Fig. 4. f4:**
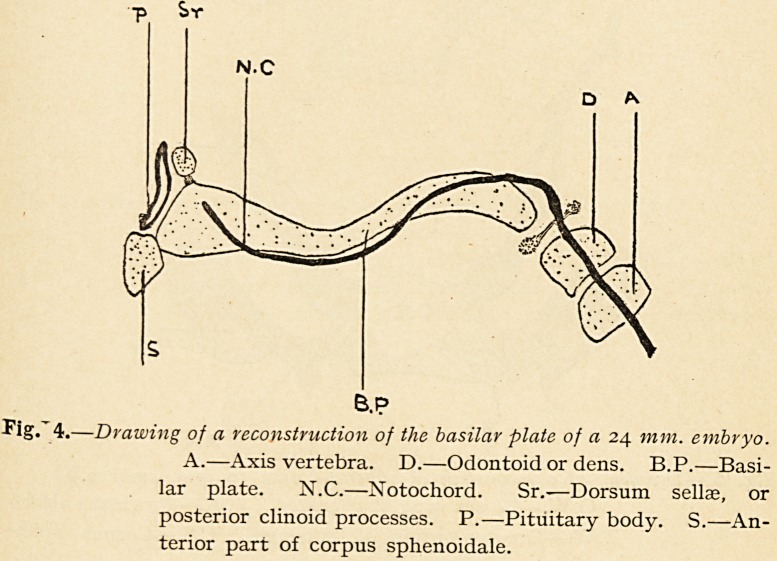


**Fig. 5. f5:**
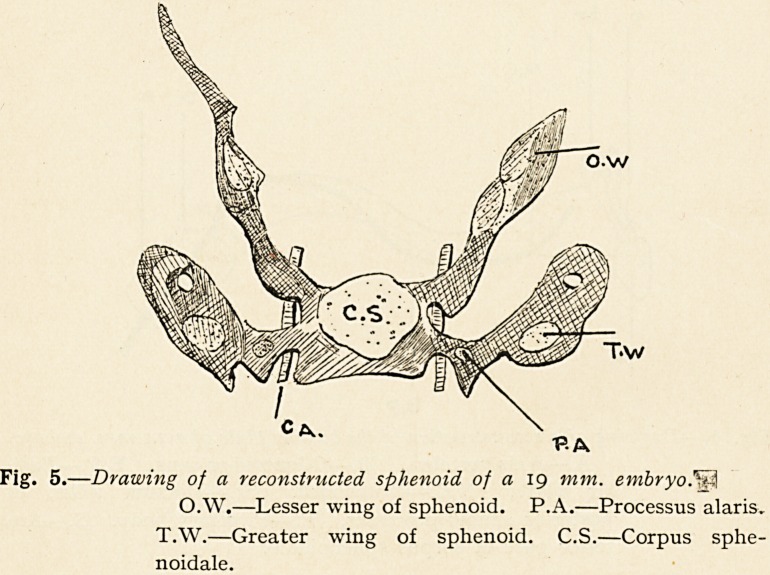


**Fig. 6. f6:**
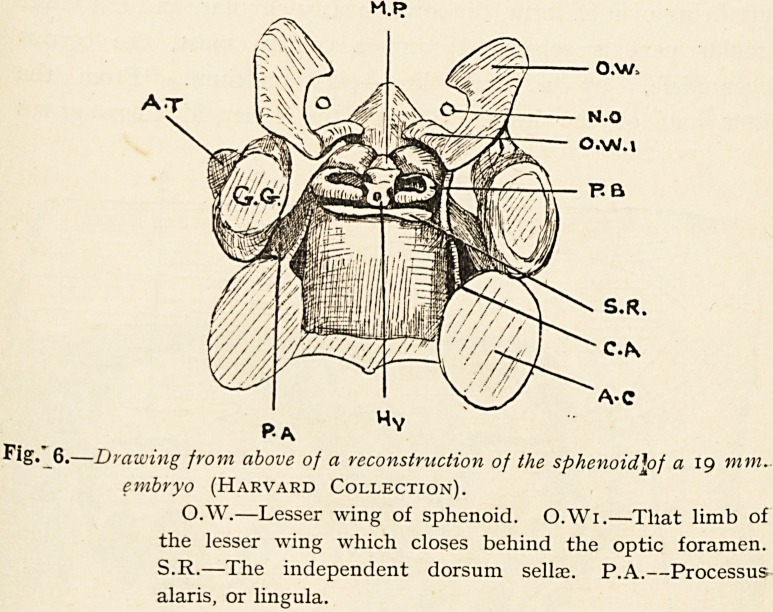


**Fig. 7. f7:**
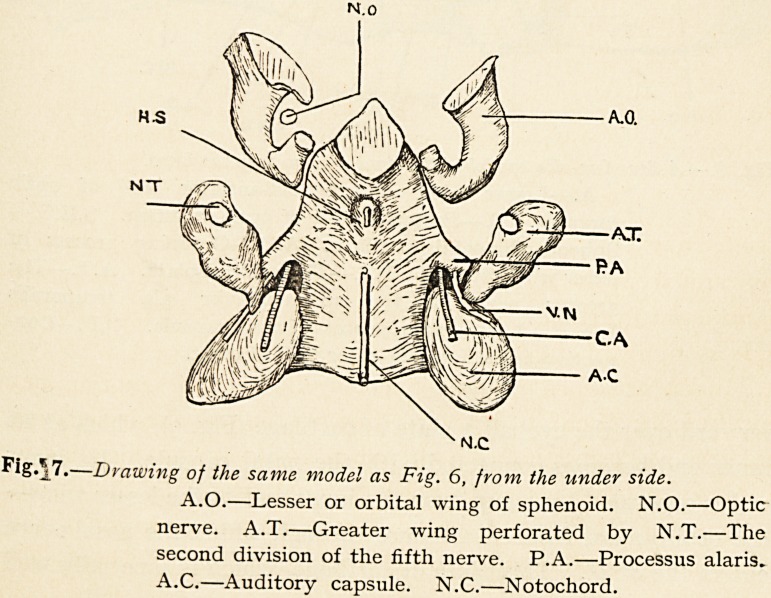


**Fig. 9. f8:**
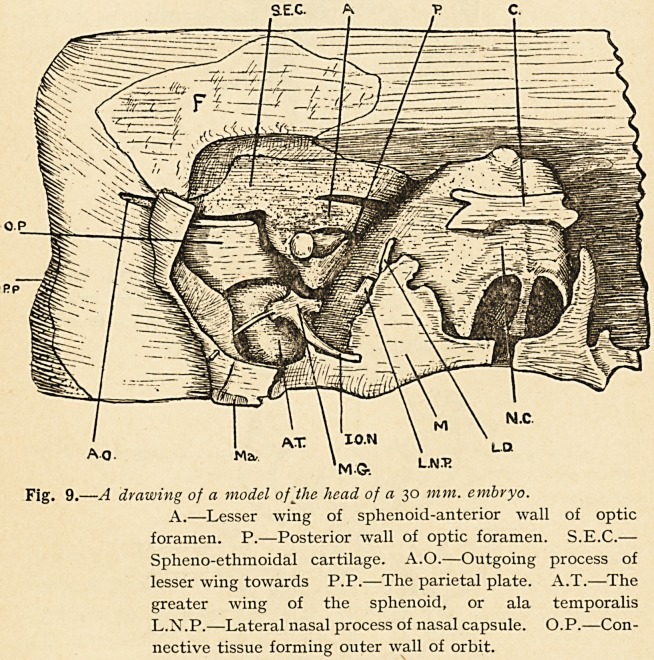


**Fig. 10. f9:**
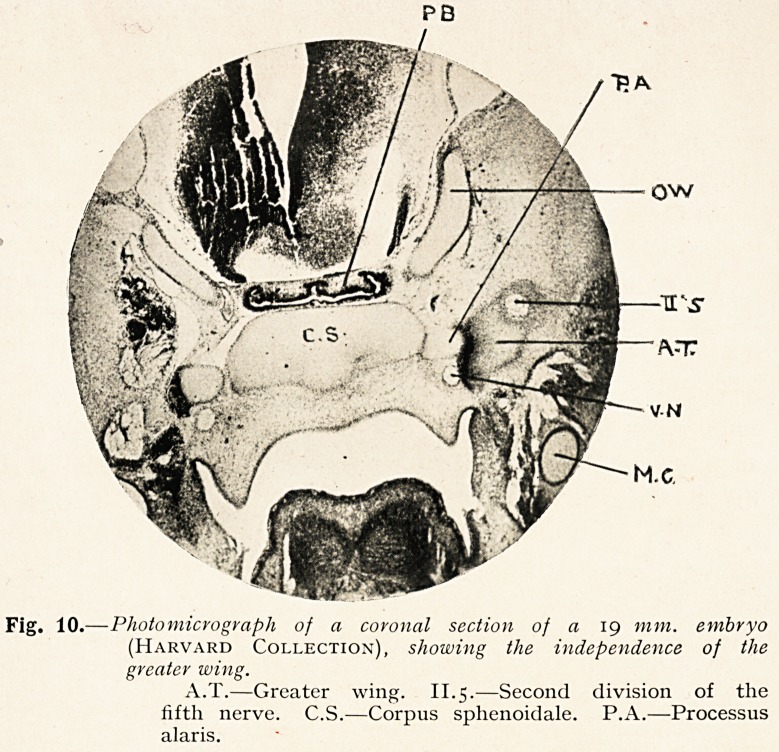


**Fig. 11. f10:**
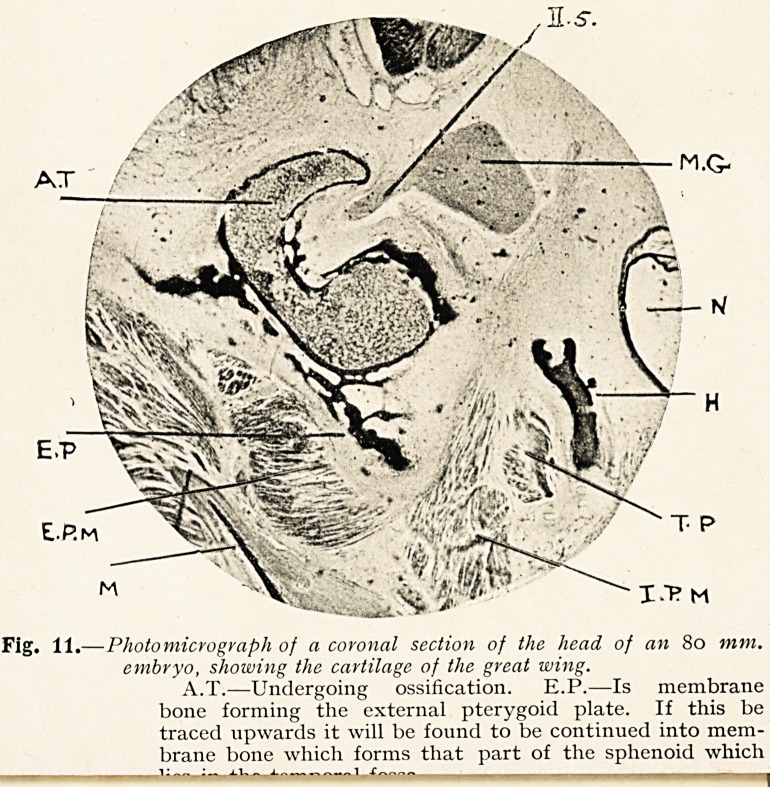


**Fig. 12. f11:**
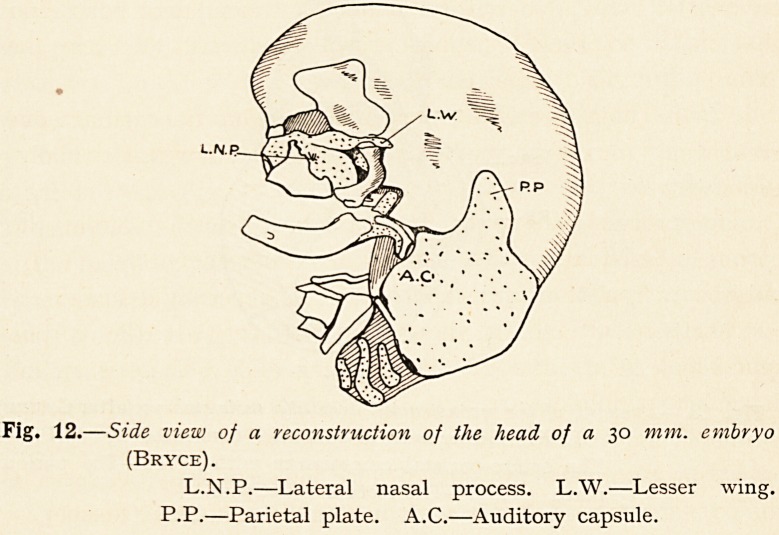


**Fig. 13. f12:**